# Chinese Species of the Genus *Pseudanaphes* Noyes & Valentine (Hymenoptera: Mymaridae) with Description of a New Species †

**DOI:** 10.3390/insects14010039

**Published:** 2022-12-31

**Authors:** Zhulidezi Aishan, Huan-Xi Cao, Hong-Ying Hu, Chao-Dong Zhu

**Affiliations:** 1 College of Life Science and Technology, Xinjiang University, Urumqi 830017, China; 2 Key Laboratory of Zoological Systematics and Evolution, Institute of Zoology, Chinese Academy of Sciences, Beijing 100101, China; 3 Xinjiang Key Laboratory of Biological Resources and Genetic Engineering, Urumqi 830017, China; 4 National Animal Collection Resource Center, Institute of Zoology, Chinese Academy of Sciences, Beijing 100101, China; 5 College of Life Sciences, University of Chinese Academy of Sciences (UCAS), Beijing 100049, China

**Keywords:** Chalcidoidea, new taxon, China, taxonomy, COI

## Abstract

**Simple Summary:**

Parasitoids of the family Mymaridae (Hymenoptera: Chalcidoidea) are difficult to collect and study, in part because of their small body size of 0.2–1.5 mm. Currently, more than 1500 species have been described and recorded. However, molecular data are rarely generated and used in relation to these small parasitoids. The genus *Pseudanaphes* is one of the smaller genera, with only five known species, for which molecular data were not available until this study. In this study, a new species of *Pseudanaphes*, *P. yadongicus* Aishan & Cao sp. nov., is separated from the known species *P. zhaoi* Lin by combining morphological and molecular data, both collected in Tibet, China. The DNA barcode and illustrations of *P. zhaoi* are also provided simultaneously. The DNA barcodes, morphological diagnosis, and a key to the world species of this genus will facilitate studies on this genus.

**Abstract:**

The fairyfly Mymaridae (Hymenoptera: Chalcidoidea) are widely distributed worldwide, but species of this family have rarely been collected and recorded from the Qinghai–Tibet Plateau. In this study, mymarids collected in Tibet, China, are identified based on morphology and molecular data. Two species of the genus *Pseudanaphes* Noyes & Valentine are treated and illustrated here, including a known species, *P. zhaoi* Lin, and a new species, *P. yadongicus* Aishan & Cao sp. nov. In addition, a key to the world species of *Pseudanaphes* (females) and DNA barcodes for *P. yadongicus* and *P. zhaoi* are provided.

## 1. Introduction

The genus *Pseudanaphes* Noyes & Valentine is one of several smaller genera in the family Mymaridae (Hymenoptera: Chalcidoidea), species of which are not commonly or numerously collected. This genus is characterized by relatively hairy fore wings and long marginal veins and was established by Noyes and Valentine (1989), who recorded an Australian species, *Pseudanaphes hirtus* Noyes & Valentine, from Titirangi, New Zealand [1]. After that, another species, *P. zhaoi* Lin, was discovered in the Palaearctic region (Shaanxi, China) [2]. Two Australian species, *P*. *lincolni* (Girault) and *P*. *particoxae* (Girault), were originally described briefly without illustrations based on a female specimen under the generic name *Polynemoidea* Girault [3,4]. New (1974) provided measurements and illustrations for these two species by examining the type specimens, and Lin (2007) transferred these two species to *Pseudanaphes* [5,6]. Later, Rehmat and Anis (2011) described another new species, *P*. *sikkimianus*, from India, based on only a single female specimen [7]. As a result, five species were known in the genus *Pseudanaphes* prior to this study. Nevertheless, the host information of *Pseudanaphes* is currently unknown, with only the type species of this genus, *P. hirtus*, being possibly associated with the eggs of weevils (Coleoptera: Curculionidae) [8].

Currently, the systematic relationship of *Pseudanaphes* with other mymarids remains unclear due to the lack of comprehensive molecular evidence and insufficient collections of this genus and possibly related groups. Usually, *Pseudanaphes* is placed in the *Anaphes* group, along with *Anaphes* Haliday, *Dorya* Noyes & Valentine, and *Erythmelus* Enock [6]. Among these genera, the species of *Pseudanaphes* are most similar to some species of *Anaphes* Haliday and can be distinguished from the latter by the rounded apex of the fore wing with a longer marginal vein and the absence of a longitudinal medina groove on the propodeum.

Species of the genus *Pseudanaphes* are rarely reported, either because they are not frequently discovered and collected in the field or because their identification is difficult. This genus also remains poorly studied in China. In this study, a new species from Tibet, China, is described, and diagnosis and illustrations are provided for a known Chinese species, *P. zhaoi* Lin. In addition, DNA barcodes of the new species and *P. zhaoi* are provided here, which are also reported for the first time for this genus.

## 2. Materials and Methods

### 2.1. Specimens Collection

All examined specimens of the two Chinese *Pseudanaphes* species were collected using Malaise traps and preserved in 99% ethanol at –20 °C until use. The holotype and one paratype of the newly described species were deposited in the Institute of Zoology, Chinese Academy of Sciences, Beijing, China (IZCAS), and the remaining paratypes and specimens of *P. zhaoi* were deposited in the Insect Collection of the College of Life Science and Technology, Urumqi, Xinjiang, China (ICXU).

### 2.2. Taxonomic Studies

Specimens were dissected and mounted on slides by using Canada balsam. All measurements were taken from slide-mounted specimens at 65×, 100×, 250×, or 400× magnification with a Leica compound microscope fitted with an eyepiece reticle. Body length, in micrometers (μm), was measured from the transverse trabecula to the metasomal apex, excluding the exserted part of the ovipositor. The remaining measurements are given either in micrometers or as ratios. Photographs were taken from slide-mounted specimens using a Nikon Ni-E system, and images were processed by Adobe Photoshop.

Abbreviations used are as follows: F1–6 = antennal funicular 1–6; POL = the shortest distance between posterior ocelli; OOL = the shortest distance between posterior ocellus and eye margin.

Terminology for morphological features follows Lin [6] and Rehmat and Anis [7].

### 2.3. DNA Extraction, Amplification, and Sequencing

Genomic DNA was extracted from 16 specimens (Table 1) using the whole body of the specimens and following the protocol of the DNeasy Blood & Tissue Kit (Qiagen, Germany). After DNA extraction, the voucher specimens were dissected and mounted on slides for further morphological examination. The voucher specimens were deposited in IZCAS.

The COI sequences were amplified using two primer pairs, Dicopus-COI-F (5′-ATCCT GGTTC ATTTT TAGGA AAT-3′) and Gonat-COI-R (5′-GCTCC NGCTA ATACW GGTAA TG-3′) and Alaptus-COI-F (5′-ATCCA GGTTC ATTTT TAGGA AAT-3′) and Gonat-COI-R (5′-GCTCC NGCTA ATACW GGTAA TG-3′), which were designed by this study based on published data of Mymaridae from the NCBI database. All PCR reactions were performed in a total volume of 30 μL, including 15 μL premix Taq polymerase (Takara, Japan), 1 μL of each forward and reverse primer, 3 μL DNA template, and 10 μL distilled water. PCR conditions followed Triapitsyn et al. [9]. Sequencing was performed in both directions.

### 2.4. Sequence Analysis

All sequences were verified by the NCBI Nucleotide Blast tool. Sequences from both directions were assembled and edited in Sequencher version 4.5 (Gene Codes Corporation, Ann Arbor, MI, USA) and aligned in BioEdit version 7.0.9.0 [10] to create the COI matrix used for distance calculations. The COI matrix was translated into amino acids in MEGA7.0 [11] to check for stop codons. A neighbor-joining (NJ) tree based on the Kimura two-parameter (K2P) distances was constructed using MEGA7.0 with 1000 bootstrap replicates to generate support values for the nodes. The COI sequences generated in this study were deposited in GenBank under accession numbers ON834699–ON834714.

## 3. Results

**Figure 1:**
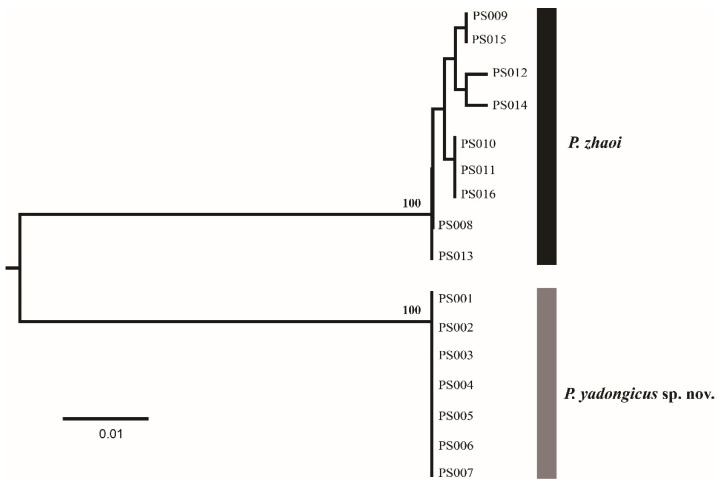
A NJ tree by K2P distances based on COI sequences from 16 *Pseudanaphes* specimens. The numbers above nodes indicate bootstrap values.

The genus *Pseudanaphes*, with one described species and one new species from China, was treated in this study. The DNA barcodes (COI) of seven females of *P. yadongicus* and nine females of *P. zhaoi* were successfully obtained. After alignment and trimming, the COI matrix without insertions or deletions, with a length of 401 base pairs, was used to obtain a NJ tree (Figure 1) by K2P distances. Based on 401 bp COI sequences, these two species were separated with high bootstrap support of 100%. The interspecific divergence (K2P distance) between these two species varied from 11.7% to 12.5%. *Pseudanaphes yadongicus* displayed no intraspecific variation, and *P. zhaoi* showed the maximum intraspecific distance at 0.8%.

### 3.1. Pseudanaphes Noyes & Valentine, 1989

*Pseudanaphes* Noyes & Valentine, 1989 [1], pp. 47–48. Type species: *Pseudanaphes hirtus* Noyes & Valentine, by original designation; Lin et al. 2007 [6], pp. 15,18,47 (list of Australia species).

Diagnosis. The genus *Pseudanaphes* Noyes & Valentine can be characterized by the following characters: female antenna with six-segmented funicle, three-segmented clava; clava large and slightly shorter than the combined length of six funiculars, and with one row of campaniform sensilla; fore wing apex evenly rounded, with venation extending at least 0.4× wing length and with disc cilia dense and with a slight brownish tinge and curved, infuscate band below proximal half of marginal vein; four-segmented tarsi. In males, antenna with 11 flagellomeres; body color and size similar to females.

**Figure 2:**
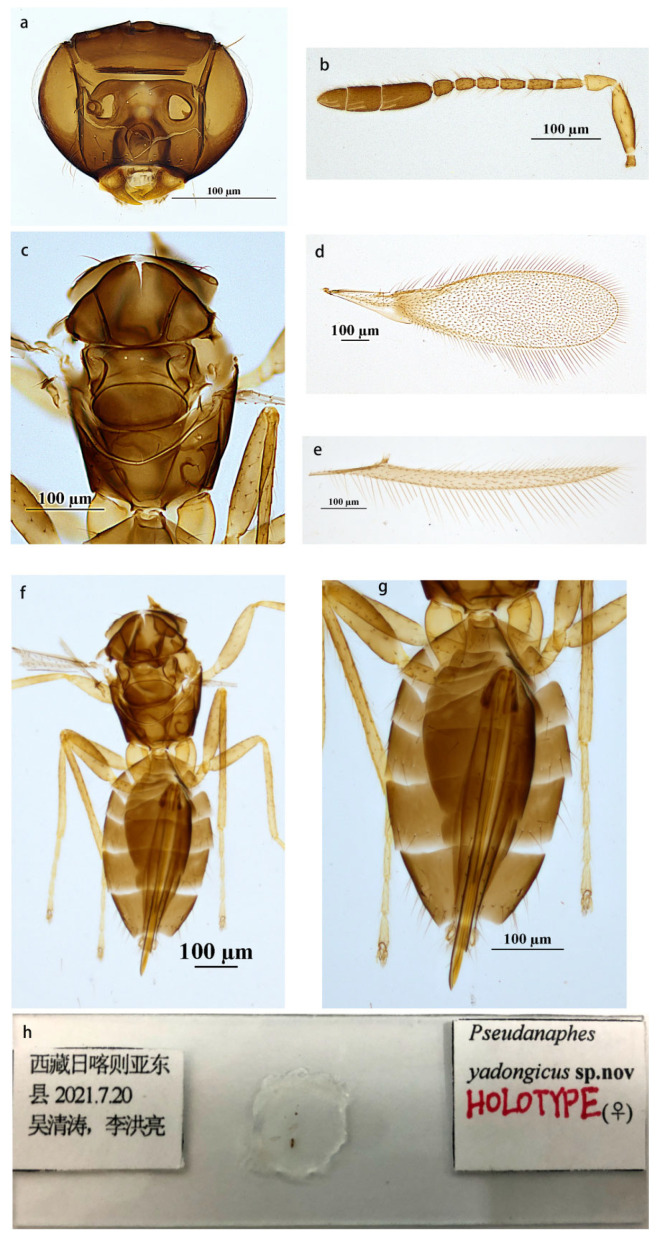
*Pseudanaphes yadongicus* sp. nov. female. (**a**) Head; (**b**) antenna; (**c**) mesosoma in dorsal view; (**d**) fore wing; (**e**) hind wing; (**f**) body in dorsal view; (**g**) metasoma in dorsal view; (**h**) holotype slide.

**Figure 3:**
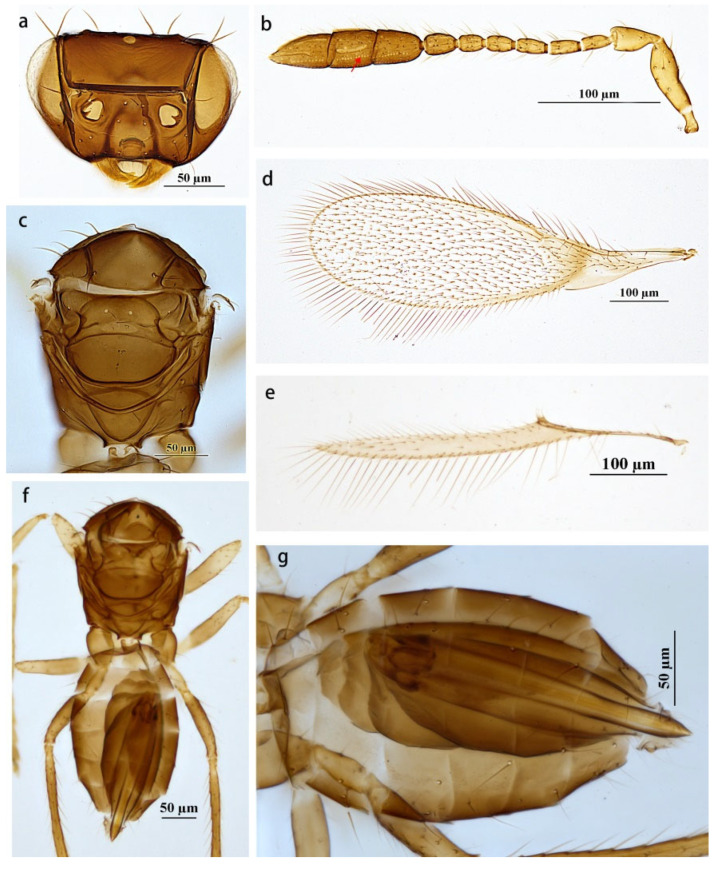
*Pseudanaphes zhaoi*. Female. (**a**) Head; (**b**) antenna; (**c**) mesosoma in dorsal view; (**d**) fore wing; (**e**) hind wing; (**f**) body in dorsal view; (**g**) metasoma in dorsal view.

The *Anaphes* group consists of four genera, *Anaphes*, *Erythmelus*, *Dorya*, and *Pseudanaphes*. The genus *Pseudanaphes* can be distinguished from the other three genera by the combination of the following characters: large and three-segmented antenna clava; fore wing apex evenly rounded, with a curved, dark mark below parastigma and a longer venation that extends at least 0.4× wing length. These characters are also useful in distinguishing species of *Pseudanapahes* from the most similar genus, *Anaphes* Haliday. Compared to the females, the species of *Anaphes* have a one- or two-segmented clava and an asymmetrical fore wing, often with an oblique row of microtrichia extending from just below the apex of the stigmal vein towards the wing apex. Lin (2007) stated that, for *Pseudanapahes*, there is no longitudinal median groove on the propodeum [6]. In contrast, a median longitudinal groove was observed on the propodeum (Figure 2c and Figure 3c) for the *Pseudanapahes* specimens examined in this study. Along with this character, the clava with one row of campaniform sensilla (Figure 3b) was suggested to be diagnostic for the genus *Pseudanapahes*.

#### 3.1.1. *Pseudanaphes yadongicus* Aishan & Cao, sp. nov.

Diagnosis. Antenna with the first claval segment the longest, apical segment the shortest, the first and second segments of clava with two longitudinal sensilla on each, and the third segment of clava with three longitudinal sensilla. Fore wing disc densely setose with setae extending to base of submarginal vein. Ovipositor long and exserted beyond apex of metasoma; ovipositor length equal to length of metasoma.

Description. Female (holotype and paratypes). Body length 1450–1470 µm. Body color brown. Antenna brown, except scape, and pedicel yellowish brown. Fore wing hyaline, slightly infuscate below submarginal vein, and with a usual, curved, infuscate band below proximal half of marginal vein. Legs yellow brown, except tarsi yellow.

Head (Figure 2a) brown and smooth, in front view wider than height (56: 36); eye 0.69× height of head (0.68–0.70×); posterior ocellus very close to eye margin, POL: OOL = 4.2. Antenna (Figure 2b) with scape about 3.75× as long as wide; pedicel about 2.0× (1.95–2.00×) as long as wide, as long as F1; the combined length of F4–F6 slightly shorter than that of three preceding ones; F2 and F3 equal in length; F5 and F6 equal in length; F6 with one longitudinal sensillum; clava about 4.77× (4.77–4.85×) as long as wide, the first segment of clava the longest, apical segment of clava the shortest, the first and second segments of clava with two sensilla on each, the third segment of clava with three sensilla.

Mesosoma (Figure 2c,f) smooth and about 0.69× length of metasoma. Lateral plate of pronotum with six setae on each, midlobe of mesoscutum with two setae, each side lobe with one, and each axilla with one seta; scutellum without setae; propodeum with a pair of setae on both sides. Fore wing (Figure 2d) about 3.81× as long as wide, disc with a slight brownish tinge and a curved, infuscate band below proximal half of marginal vein, and infuscate below submarginal vein; densely setose, with setae extending nearly to the wing base; longest marginal seta 0.49× (0.48–0.52×) greatest width of wing. Hind wing (Figure 1e) 21× as long as wide; longest marginal seta 2× greatest width of wing.

Metasoma (Figure 2g) smooth. Ovipositor originating from about the level of the second metasomal tergite and occupying about 0.99× length of metasoma, exserted beyond apex of metasoma by 0.13× (0.13–0.14×) its own length, 2.0× (1.9–2.0×) as long as mesotibia.

Measurements of the holotype (Figure 2h). Antenna: scape: 150: 40; pedicel: 60: 30; F1: 60; F2: 50; F3: 50; F4: 45; F5: 40; F6: 40; clava: 260. Mesosoma: 500; fore wing: 1640: 430; longest marginal seta: 210. Hind wing: 1260: 60; longest marginal seta: 20. Metasoma: 720; ovipositor: 715.

MALE. Unknown.

Etymology. The species is named after the Yadong county of Tibet, China, where the type material was collected.

Type material. Holotype, female, China, Tibet, Yadong County, 20 July 2021, Qing-Tao Wu and Hong-Liang Li (IZCAS, IOZ(E) 221465). Paratypes: one female, same data as the holotype (IZCAS, IOZ(E) 221466); five females, same data as the holotype (ICXU).

Biology. Unknown.

Distribution. China (Tibet).

Comments. Females of *P. yadongicus* can be recognized by the combination of characters described in the diagnosis. Accordingly, the females of this species resemble those of *P. lincolni* (Girault). In females, *P. yadongicus* differs from the latter species in antenna color, relative length of F1 and the pedicel, relative length of the three claval segments, and relative length of the extended ovipositor. Females of *P. yadongicus* have brown antenna with yellowish brown scape and pedicel, F1 and pedicel of subequal length, the longest F1 of the funicle, and the longest first segment of the clava (Figure 2b); they have an ovipositor notably exserted beyond the apex of the metasoma (Figure 2g), whereas, in comparison to *P. yadongicus*, females of *P. lincolni* have black antennae, F1 longer than pedicel, F2 and pedicel of subequal length, the longest F2 of the funicle, and the ovipositor not notably exserted beyond the apex of the metasoma as in the former species.

#### 3.1.2. *Pseudanaphes zhaoi* Lin, 1997

*Pseudanaphes zhaoi* Lin 1997 [2], pp. 98–100. Type locality: Shaanxi, China.

Diagnosis. Antenna (Figure 3b): F5 the shortest funicular segment; the first and second claval segments subequal in length, apical segment longer than each of them; the first segment of clava without sensilla, the second segment of clava with two longitudinal sensilla, and the third segment of clava with three longitudinal sensilla. Mesosoma (Figure 3c) about 0.77× length of metasoma. Fore wing (Figure 3d) disc with a slight brownish tinge and a curved, infuscate band below proximal half of marginal vein and slightly infuscate below submarginal vein; densely setose, with setae reaching about half length of submarginal vein. Hind wing (Figure 3e) 16.92× as long as wide; longest marginal seta 3.08× greatest width of wing. Metasoma (Figure 3f): ovipositor (Figure 3g) not or barely exserted beyond the metasomal apex.

Material examined. Six females, China, Tibet, Yadong County, 20 July 2021, Q. Wu (ICXU); three females, Yadong County, 1 July 2020, Qing-Tao Wu and Hong-Liang Li (ICXU).

Distribution. China (Tibet, Shaanxi).

Biology. Unknown.

**Figure 4:**
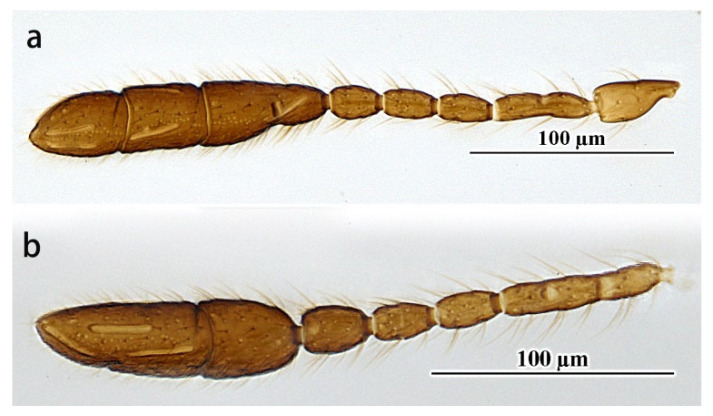
*Pseudanaphes zhaoi*. Female. (**a**), (**b**) Antenna (without scape and pedicel).

Comments. The specimens studied here differ from the holotype in the following characters: F6 with one longitudinal sensillum; fore wing 3.25–3.58× as long as wide, with longest marginal seta 0.42–0.52× greatest width of the fore wing; ovipositor 1.39–1.42× as long as the mesotibia. In addition, for one specimen of the material examined above, the apical two claval segments, F2 and F3, are coalesced (Figure 4a,b). These characteristics fall within the range of intraspecific variations.

### 3.2. Key to Species of Pseudanaphes (Female)

urn:lsid:zoobank.org:act:7B041410-9F8A-4AB2-A693-BB83DA19F0FF

1. The first claval segment longer than the other two claval segments…2
- The first claval segment shorter than at least one of the other two claval segments…3
2. Ovipositor notably exserted beyond apex of metasoma; clava at most 4.85× as long as wide, apical claval segment the shortest…*P. yadongicus* Aishan & Cao, sp. nov.
- Ovipositor not exserted beyond apex of metasoma; clava 5.91× as long as wide, second claval segment the shortest…*P. lincolni* (Girault)
3. Metasoma with anterior scutellum with reticulate sculpture near posterior margin…P. hirtus Noyes & Valentine
- Metasoma smooth, without reticulate sculpture…4
4. Mesothorax golden; first segment of clava the shortest, clava 3.42× as long as wide…*P. particoxae* (Girault)
- Mesothorax brown to dark brown; first segment of clava not the shortest, clava at least 3.9× as long as wide…5
5. F5 and F4 subequal in length, F4 without longitudinal sensilla; anterior scutellum without setae; fore wing 3.33× as long as wide…*P. sikkimianus* Rehmat & Anis
- F5 distinctly shorter than F4, F4 with one longitudinal sensillum; anterior scutellum with one pair of setae; fore wing 2.85× as long as wide…*P. zhaoi* Lin

## 4. Discussion

Almost all species of Mymaridae are important parasitoids of the eggs of a wide variety of insect pests [12]. Currently, more than 1500 species in 115 genera of Mymaridae are known worldwide [2,9]. Although a total of 31,839 COI sequences of Mymaridae have been recorded in GenBank (National Center for Biotechnology Information, NCBI), most of them were sequenced from unnamed species with a high repeat rate of 80%, and 673 of them were sequenced from only thirteen named species of *Anaphes* Haliday, *Anagrus* Haliday, *Cosmocomoidea* Howard, and *Ooctonus* Haliday [13,14,15,16,17,18,19,20]. In this study, COI primers for the genus *Pseudanaphes* and COI sequences of *Pseudanaphes* were developed and generated for the first time, which provide a reference for studies on the family Mymaridae. The results of molecular analysis support the species delimitation of these two *Pseudanaphes* species, *P. yadongicus* and *P. zhaoi*. In addition, it is suggested that the antennal clava with one row of campaniform sensilla could be used as a diagnosis for the genus *Pseudanaphes*. This will facilitate the identification and discovery of *Pseudanaphes* species or even other mymarids.

## Figures and Tables

**Table 1:** Information on sequenced specimens with GenBank accession number of COI.

Code	Species	Sex	Primer Pair	Accession Number
PS001 PS002 PS003 PS004 PS005 PS006 PS007 PS008 PS009 PS010 PS011 PS012 PS013 PS014 PS015 PS016	*P. yadongicus* *P. yadongicus* *P. yadongicus* *P. yadongicus* *P. yadongicus* *P. yadongicus* *P. yadongicus* *P. zhaoi* *P. zhaoi* *P. zhaoi* *P. zhaoi* *P. zhaoi* *P. zhaoi* *P. zhaoi* *P. zhaoi* *P. zhaoi*	female female female female female female female female female female female female female female female female	Dicopus-COI-F and Gonat-COI-R Alaptus-COI-F and Gonat-COI-R Dicopus-COI-F and Gonat-COI-R Dicopus-COI-F and Gonat-COI-R Dicopus-COI-F and Gonat-COI-R Dicopus-COI-F and Gonat-COI-R Alaptus-COI-F and Gonat-COI-R Dicopus-COI-F and Gonat-COI-R Dicopus-COI-F and Gonat-COI-R Dicopus-COI-F and Gonat-COI-R Dicopus-COI-F and Gonat-COI-R Dicopus-COI-F and Gonat-COI-R Dicopus-COI-F and Gonat-COI-R Alaptus-COI-F and Gonat-COI-R Alaptus-COI-F and Gonat-COI-R Alaptus-COI-F and Gonat-COI-R	ON834699 ON834700 ON834701 ON834702 ON834703 ON834704 ON834705 ON834706 ON834707 ON834708 ON834709 ON834710 ON834711 ON834712 ON834713 ON834714

## Data Availability

DNA sequence data are available on GenBank under accession numbers ON834699–ON834714. Other data of this research were deposited in the Institute of Zoology, Chinese Academy of Sciences, Beijing, China.
